# Human Hsp40 proteins, DNAJA1 and DNAJA2, as potential targets of the immune response triggered by bacterial DnaJ in rheumatoid arthritis

**DOI:** 10.1007/s12192-013-0407-1

**Published:** 2013-02-14

**Authors:** Agnieszka Kotlarz, Stefan Tukaj, Konrad Krzewski, Elzbieta Brycka, Barbara Lipinska

**Affiliations:** 1 Department of Biochemistry, University of Gdansk, Wita Stwosza 59, 80-308 Gdansk, Poland; 2 Department of Plant Physiology and Biotechnology, University of Gdańsk, Wita Stwosza 59, 80-308 Gdańsk, Poland; 3 Department of Molecular Virology, University of Gdansk and Medical University of Gdansk, Kladki 24, 80-822 Gdansk, Poland; 4 National Institute of Allergy and Infectious Diseases, National Institutes of Health, Rockville, MD USA

**Keywords:** Anti-Hsp40 autoantibodies, Hsp40 in rheumatoid arthritis, Cross-reactivity of anti-Hsp40 antibodies, Anti-DNAJ monoclonal antibodies, Molecular mimicry

## Abstract

Hsp40 proteins of bacterial and human origin are suspected to be involved in the pathogenesis of rheumatoid arthritis (RA). It has been shown that sera of RA patients contain increased levels of antibodies directed to bacterial and human Hsp40s. The aim of this work was to explore immunological similarities between the bacterial (DnaJ) and human (DNAJA1 and DNAJA2) Hsp40 proteins in relation to their possible involvement in the RA. Using polyclonal antibodies directed against a full-length DnaJ or its domains, against DNAJA1 and DNAJA2, as well as monoclonal anti-DnaJ antibodies, we found immunological similarities between the bacterial and human Hsp40s. Both ELISA and Western blotting showed that these similarities were not restricted to the conserved J domains but were also present in the C-terminal variable regions. We also found a positive correlation between the levels of the anti-DnaJ and anti-DNAJA1 antibodies in the sera of RA patients. This finding supports the molecular mimicry hypothesis that human Hsp40 could be the targets of antibodies originally directed against bacterial DnaJ in RA.

**Electronic supplementary material:**

The online version of this article (doi:10.1007/s12192-013-0407-1) contains supplementary material, which is available to authorized users.

## Introduction

Heat shock proteins (Hsps) are a family of evolutionarily conserved proteins, which play an important role in cell physiology under the normal and stress conditions. At times of cellular stress, including infection and chronic inflammation, such as present in autoimmune diseases, the expression of Hsps is markedly elevated (reviewed in van Eden et al. 2005; Borges et al. 2012). Hsps are also a group of major bacterial antigens (Albani et al. 1995; reviewed in Borges et al. 2012), and the conservation of their structure from bacteria to man, as well as high immunogenicity, makes them attractive targets for investigation in the area of autoimmunity. In this area, the Hsps of the Hsp60 and Hsp70 families were the ones most extensively studied, especially since the discovery that the T cells isolated from the rats with adjuvant-induced arthritis were responding to mycobacterial Hsp60 (van Eden et al. 1988; reviewed in van Eden et al. 2005). To the contrary, the research on Hsp40 involvement in autoimmune diseases has been less extensive, in spite of the fact that Hsp40 is probably the largest Hsp family in humans, with at least 50 members (reviewed in Kampinga et al. 2009).

The model representative of the Hsp40 family is the *Escherichia coli* DnaJ protein, composed of 375 amino acids in four domains. The amino-terminal 75 residues of DnaJ constitute an evolutionarily highly conserved motif, the J domain, which together with the adjacent region, rich in glycine and phenylalanine, is essential for DnaJ’s interactions with Hsp70 chaperone. The third domain, rich in cysteine residues, together with the least conserved C-terminal region, functions to bind substrate proteins (Qiu et al. 2006; reviewed in Kampinga et al. 2009). Of human Hsp40, the DNAJB1 (Hdj1), DNAJA1 (Hdj2), and DNAJA2 (Hdj3) proteins are best characterized (Terada and Mori 2000; reviewed by Sterrenberg et al. 2011). DNAJA1 and DNAJA2, belonging to the class A of Hsp40, bear the highest structural similarity to the bacterial DnaJ and possess all the domains characteristic for DnaJ, while DNAJB1 does not have the cysteine-rich domain (Cheetham and Caplan 1998; Kampinga et al. 2009).

Bacterial and human Hsp40 are suspected to participate in the autoimmune response during pathogenesis of rheumatoid arthritis (RA) and juvenile idiopathic arthritis (JIA). The presence of elevated levels of antibodies against the *E. coli* DnaJ has been shown in RA (Albani et al. 1994, 1995; Chukwuocha et al. 1999; Tukaj et al. 2010a), with especially high response to the conserved J domain of DnaJ (Albani et al. 1995; Tukaj et al. 2010a). Additionally, an overexpression of human Hsp40s and significantly increased levels of the anti-DNAJA1 and anti-DNAJA2 antibodies have been found, respectively, in the synovial tissue and sera of RA patients (Kurzik-Dumke et al. 1999; Tukaj et al. 2010a). Initially, a “molecular mimicry hypothesis” has been proposed, suggesting that the immune response directed against the bacterial DnaJ protein cross-reacts with the human homologous protein(s) and promotes development of RA; an infection with various bacterial species could trigger the response, since DnaJ is highly conserved among bacteria (Albani et al. 1995; Albani and Carson 1996). Indeed, bacterial infection is considered as one of the possible factors promoting development of RA (reviewed in Lundberg et al. 2010). Later on, another aspect of the Hsp40s role in inflammatory diseases emerged, showing an immunomodulatory role of Hsp40s in downregulating immune response. It has been demonstrated that T cells from patients with JIA respond differentially to peptides derived from bacterial and human Hsp40s and that regulatory T cells, induced by a peptide derived from a human Hsp40, downregulate proliferation of synovial fluid mononuclear cells of JIA patients (Massa et al. 2007). Our previous study showed that DnaJ and human Hsp40 proteins inhibited proliferation of T cells of the RA patients and had an immunomodulatory effect on cytokine secretion by the patients’ lymphocytes (Tukaj et al. 2010a). Although the exact role of bacterial and human Hsp40s in the autoimmune response requires further elucidation, it is clear that those proteins have a relevance in the clinical setting, especially since Hsp40s are considered as potential targets for anti-inflammatory therapy in JIA (Massa et al. 2007) and RA (Tukaj et al. 2010a).

Previously, we have shown a significant immunological similarity between bacterial DnaJ and human DNAJB1, not restricted to the conserved J domains of the proteins (Krzewski et al. 2003). The aim of this work was to further investigate the immunological similarities among the bacterial and human Hsp40s and focus on the DNAJA1 and DNAJA2, the human Hsp40s best structurally matched with DnaJ. We tested cross-reactivity of the bacterial and human Hsp40s with the monoclonal antibodies, recognizing different and defined DnaJ epitopes, and with polyclonal antibodies against full-length DnaJ, DnaJ N-terminal domain (N-DnaJ), DnaJ C-terminal domain (C-DnaJ), DNAJA1, and DNAJA2. We also assayed the humoral anti-Hsp40 response in RA patients and analyzed correlation between the bacterial and human anti-Hsp40 antibody levels.

## Results and discussion

### Polyclonal antibodies against DnaJ, N-DnaJ, and C-DnaJ react with human DNAJA1 and DNAJA2

**Fig. 1:**
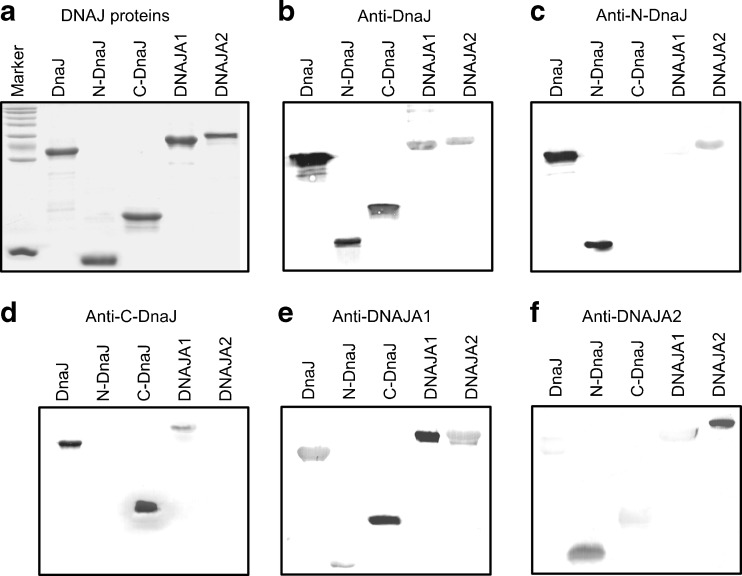
Cross-reactions between anti-DNAJ(Hsp40) polyclonal antibodies and DNAJ(Hsp40) proteins tested by Western blotting. The DnaJ, N-DnaJ (DnaJΔ107-375), C-DnaJ (DnaJΔ1-199), DNAJA1, and DNAJA2 recombinant proteins were overproduced in *E. coli* cells, purified, and resolved by SDS-PAGE. Of each Hsp40 protein, 2.5 μg has been loaded onto the gel (**a**). The polyclonal antibodies indicated above the panels were used in Western blotting as the primary antibodies (**b**–**f**)

We purified recombinant bacterial (*E. coli* DnaJ, N-DnaJ, C-DnaJ) and human (DNAJA1 and DNAJA2) Hsp40 proteins, overproduced in bacterial cells (Fig. 1a), and used them as antigens to obtain rabbit polyclonal antibodies. Cross-reactivity of these antibodies with the Hsp40 proteins was tested by Western blotting technique and ELISA tests.

The immunoblotting results showed that the anti-DnaJ antibodies were able to cross-react with both DNAJA1 and DNAJA2 (Fig. 1b). The anti-N-DnaJ antibodies reacted with DNAJA2 but not with DNAJA1 (Fig. 1c). Conversely, the anti-C-DnaJ antibodies reacted with DNAJA1 but not with DNAJA2 (Fig. 1d). These results indicate immunological similarity between DnaJ, and human DNAJA1 and DNAJA2, with DNAJA1 bearing a higher similarity to the C-terminal part of DnaJ and DNAJA2 to the N-terminal domain. This conclusion was confirmed further by the fact that the anti-DNAJA1 serum reacted strongly with C-DnaJ protein and weakly with N-DnaJ (Fig. 1e), while the anti-DNAJA2 antibodies reacted well with N-DnaJ but very weakly with C-DnaJ (Fig. 1f). The identity at the amino acid level between DnaJ and DNAJA1 or DNAJA2 is 38 or 39 %, respectively. Surprisingly, we noticed a very weak reaction of anti-DNAJA1 serum with DNAJA2 in spite of 54 % identity at the amino acid level between DNAJA1 and DNAJA2 (Fig. 1e). In parallel, the anti-DNAJA2 antibodies reacted very weakly or not at all with DNAJA1 (Fig. 1f and results not shown). The latter finding is in agreement with the results of Terada and Mori (2000), who showed the lack of cross-reactivity of the anti-Hdj3 (DNAJA2) antibodies with Hdj2 (DNAJA1) in immunoblotting. This also shows that the degrees of sequence identity and immunological similarity may differ. It should be noted that the anti-DNAJA1 serum recognized the full-length DnaJ (Fig. 1e).

**Fig. 2:**
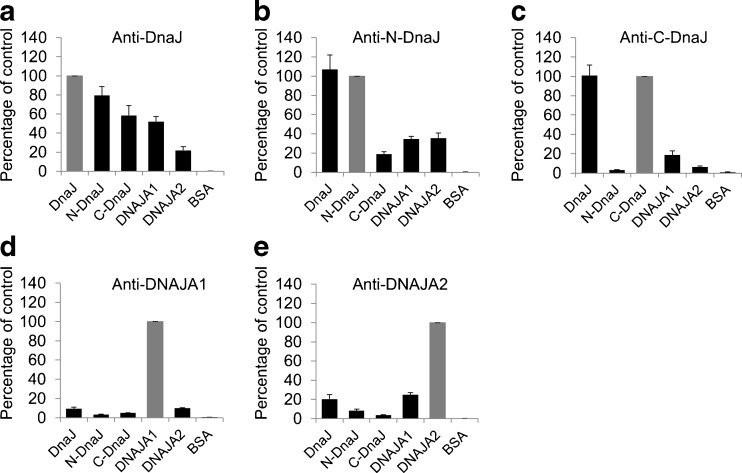
Cross-reactions between anti-DNAJ(Hsp40) polyclonal antibodies and DNAJ(Hsp40) proteins tested by ELISA. The reactivity of polyclonal anti-DnaJ (**a**), anti-N-DnaJ (**b**), anti-C-DnaJ (**c**), anti-DNAJA1 (**d**), and anti-DNAJA2 (**e**) with purified non-denatured DNAJ antigens was tested as described in “Materials and methods.” Data obtained for the antigens at 40 μg per milliliter are presented as the mean ± SEM of three independent experiments and expressed as a percentage of control (i.e., reactivity with the antigen used to raise a given antibody; indicated as the *gray bar*)

The results obtained by the Western blotting technique, in which only linear epitopes were recognized, were confirmed by the ELISA tests in which non-denatured antigens were used. The anti-DnaJ antibodies cross-reacted with both DNAJA1 and DNAJA2 (Fig. 2a). The anti-N-DnaJ antibodies reacted well with both DNAJA1 and DNAJA2 (Fig. 2b), while in the immunoblots, the reaction with DNAJA1 was almost non-detectable. The reason why we could observe the reaction with DNAJA1 here but not in the immunoblots could be due to the presence of the native antigens and thus of conformational epitopes and/or the higher sensitivity of the ELISA test compared to Western blotting. It should be noted that the J domains of DnaJ and DNAJA1 or DNAJA2 bear about 50 % identity (as calculated for the residues 1–77, 3–78, and 5–80 of DnaJ, DNAJA1, and DnaAJA2, respectively). In the case of anti-C-DnaJ antibodies, a cross-reaction with DNAJA1 and a minimal binding to DNAJA2 was observed (Fig. 2c), which was consistent with the immunoblotting results. Like in immunoblotting, anti-DNAJA1 and anti-DNAJA2 showed very weak cross-reactivity with DNAJA2 and DNAJA1, respectively (Fig. 2d, e). In ELISA tests, these antibodies showed poorer reaction with bacterial proteins than in Western blotting. This could be due to inaccessibility of the linear epitopes which could be exposed only after a denaturation process takes place during SDS-PAGE in Western blotting experiments.

Taken together, all the above results show a significant immunological similarity between DnaJ and DNAJA1/DNAJA2 proteins, which in the case of DNAJA1 was found not only in the conserved J domain but also in the poorly conserved C-terminal region. Additionally, a very low immunological similarity was found between DNAJA1 and DNAJA2 proteins, suggesting that these antigens induce different immune responses.

### The monoclonal antibodies recognizing DnaJ epitopes react with DNAJA1 and DNAJA2

**Fig. 3:**
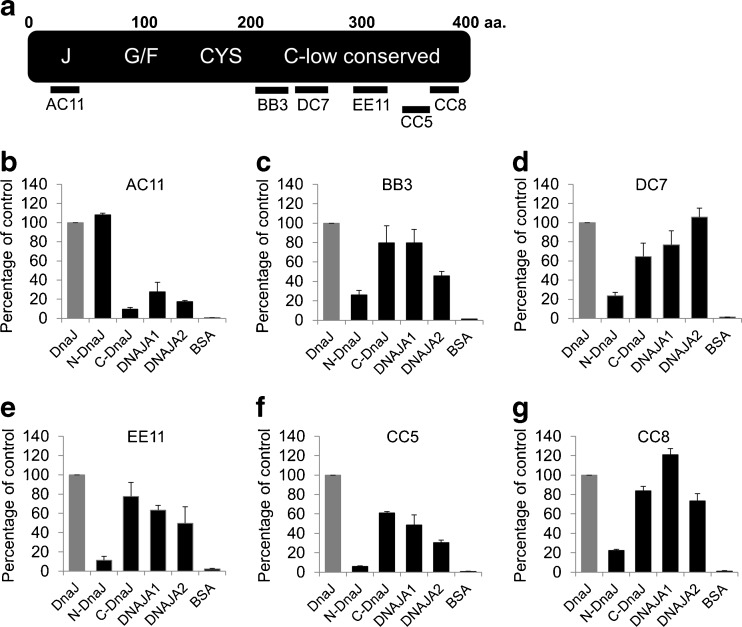
Reactivity of anti-DnaJ monoclonal antibodies (mAbs) with human DNAJA proteins. Schematic outline of the DnaJ linear structure and localization of the epitopes recognized by the mAbs are presented in **a**. The reactivity of six mAbs (**b**–**g**) with purified non-denatured DNAJ(Hsp40) antigens was assayed by ELISA test as described in “Materials and methods.” Data obtained for the antigens at 40 μg per milliliter are presented as the mean ± SEM of three independent experiments and expressed as a percentage of the reactivity with DnaJ control (*gray bar*)

To further characterize response of human Hsp40 proteins to anti-DnaJ antibodies, we used a set of six anti-DnaJ monoclonal antibodies (mAbs), whose epitopes have been characterized previously (Krzewski et al. 2003); localization of these epitopes in DnaJ protein is shown schematically in Fig. 3a. The mAb AC11 epitope is localized in a highly conserved region of the J domain while the other epitopes (BB3, DC7, EE11, CC5, and CC8) are placed in the weakly conserved C-terminal region. It has been previously shown (Krzewski et al. 2003) that all these mAbs bind more efficiently to the native DnaJ compared to the denatured protein. In ELISA tests, the AC11 mAb reacted with both DNAJA1 and DNAJA2 (Fig. 3b). Surprisingly, all the mAbs directed to the C-terminal region of DnaJ (Fig. 3a) recognized both human Hsp40s with high efficiency (Fig. 3c–g). These results indicate that, despite a low identity (not exceeding 20 %) of amino acid sequence among the C-terminal regions of DnaJ and DNAJA1/DNAJA2, they do have some similar epitopes (the amino acid residues used to calculate the identity were 198–373 for DNAJA1 and 206–381 for DNAJA2). These epitopes are most probably of the conformational type, since the mAbs used here preferentially bind to the native DnaJ, and also alignment of the mAbs epitope consensus sequences with the DNAJA1 and DNAJA2 amino acid sequences did not reveal any putative linear epitopes (results not shown). These results further confirm that the anti-DnaJ antibodies can recognize human DNAJA1 and DNAJA2, and the recognition is not restricted to the conserved J domains. They also suggest that the C-terminal domains of DnaJ and DNAJA1/DNAJA2 have a similar three-dimensional structure. To date, the structures of the C-terminal, substrate-binding regions of these proteins have not been solved. Within the Hsp40 family of proteins, the C-terminal domains of the yeast Sis1 (Sha et al. 2000) and human Hdj1 (DNAJAB1) protein (Suzuki et al. 2010) have been crystallized and characterized.

The major conclusion that the anti-DnaJ antibodies can recognize human DNAJA1 and DNAJA2, and the recognition is not restricted to the conserved J domain, supports the “molecular mimicry” hypothesis. This hypothesis states that in RA, human Hsp40 protein(s) may be the targets of immune response induced by a bacterial DnaJ antigen (Albani et al. 1995; Albani and Carson 1996). Our previous observations that sera derived from RA patients contain significantly elevated levels of anti-DnaJ, anti-DNAJA1, and anti-DNAJA2 (but not anti-DNAJB1) antibodies (Tukaj et al. 2010a, b) are also consistent with the mimicry hypothesis. In this study, using a different set of the RA patient sera, we obtained similar results (Table S1, Supplementary data). Furthermore, we found a positive correlation between the levels of the anti-DnaJ and anti-DNAJA1 antibodies in the RA sera (Table 1). Since DNAJA1 and DNAJA2 are modified by farnesylation in humans (Terada and Mori 2000) but not in the bacterial systems, we also purified and tested the overproduction and farnesylation of these proteins in the baculovirus eukaryotic expression system. We did not find any significant differences in the reactivity of the farnesylated and non-farnesylated proteins with the RA sera (Table S1, Supplementary data). In the case of the farnesylated proteins, the positive correlation between the anti-DnaJ and anti-DNAJA1 responses was also found (Table 1). The observed correlations indicate that in RA patients, the anti-DnaJ response may be directed against the DNAJA1 protein.

**Table 1:** Correlation of the level of the anti-DnaJ antibodies (IgG) with the levels of the anti-DNAJA1/2 autoantibodies (IgG) in the sera of the rheumatoid arthritis (RA) patients

	DnaJ (2.5)	DnaJ (0.15)
DNAJA1		
(2.5)	*0.631*, *p < 0.001*	
(0.15)		*0.847*, *p < 0.001*
DNAJA2		
(2.5)	−0.151, *p* = 0.333	
(0.15)		−0.150, *p* = 0.338
DNAJA1^f^		
(2.5)	*0.442*, *p = 0.003*	
(0.15)		*0.33*, *p = 0.029*
DNAJA2^f^		
(2.5)	0.176, *p* = 0.259	
(0.15)		0.161, *p* = 0.302

The levels of the antibodies were assayed by ELISA test in the sera of 43 RA patients (presented in Table S1, Supplementary data) as described in “Materials and methods,” using antigens at the concentrations (in micrograms per milliliter) indicated in parentheses. DNAJA1^f^ and DNAJA2^f^ are the farnesylated proteins produced in the baculovirus system. The other proteins were produced in the bacterial system. The correlations between the antibody levels are presented as Pearson’s coefficients (*r*). The statistically significant values are shown in italics

The question whether the postulated cross-reaction between the anti-DnaJ antibodies and human Hsp40s may lead to pathological consequences remains open. There is increasing evidence that Hsps may function as factors downregulating immune response. It has been shown that the T cells immunoreactive to various types of self-Hsps display regulatory properties (van Eden et al. 2005; Wieten et al. 2007, 2010; reviewed in Borges et al. 2012). The regulatory role of Hsp40s encouraged the idea that they may be potential targets for designing new anti-inflammatory therapies (Massa et al. 2007; Tukaj et al. 2010a). Furthermore, a peptide derived from the J domain of DnaJ has been successfully applied in clinical trials of immunotherapy of RA patients (Koffeman et al. 2009). We believe that the information presented in this work increases our understanding of the anti-Hsp40 immune response and may be useful in designing new anti-inflammatory therapies.

## Materials and methods

### Protein overproduction and purification

The DnaJ, N-DnaJ (DnaJΔ107-375), C-DnaJ (DnaJΔ1-199), DNAJA1, and DNAJA2 recombinant proteins were overproduced in *E. coli* cells and purified as described previously (Krzewski et al. 2003; Tukaj et al. 2010a). To obtain the DNAJA1 and DNAJA2 proteins modified by farnesylation, the insect cells (*Spodoptera frugiperda* Sf9) and baculovirus expression system Bac-To-Bac (Invitrogen) were used, according to the manufacturer’s instruction. We constructed the pFastBac1/DNAJA1 and pFastBac1/DNAJA2 transfer plasmids, encoding the His_6_-DNAJA1 and His_6_-DNAJA2 proteins, respectively. The plasmid pFastBac1/DNAJA1 was derived from the pMal-c2x/hdj2 (Kanazawa et al. 1997) by excising the *Ecl*136-*Xba*I fragment and inserting it into a pFastBac1 vector. The plasmid pFastBac1/DNAJA2 was constructed by excising the *BamH*I–*Pst*I fragment of the pVL1393/hdj3 plasmid (Terada et al. 1997) and inserting it into a pFastBac1 vector. Other procedures were as described in the manufacturer’s manual. Farnesylation of His-DNAJA1/2 proteins was enhanced by exogenously adding a mevalonate precursor, mevalonolactone (Sigma), to the Sf-900 II SFM medium, at 5 mM. The His-DNAJA1/2 proteins produced in insect cells were purified from the cytoplasmic fraction using a Ni-NTA agarose (Qiagen), according to the manufacturer’s manual.

### Polyclonal and monoclonal antibodies

The polyclonal antisera were raised by immunizing rabbits with the purified recombinant Hsp40 proteins according to the method described by Szewczyk and Harper (1994) and tested by Western blotting with appropriate antigens. The mouse monoclonal antibodies AC11, BB3, CC8, EE11, DC7, and CC8 were generated and characterized as described by Krzewski et al. (2003).

### Protein electrophoresis and Western blotting

Hsp40 proteins were resolved in 15 % polyacrylamide gels, in denaturing conditions. Western blotting was performed, using the polyclonal rabbit anti-Hsp40 sera as the primary antibodies and alkaline phosphatase-conjugated goat anti-rabbit immunoglobulins (Roche) as secondary antibodies, as described previously (Krzewski et al. 2003). The primary antibodies were used at the following dilutions: anti-DnaJ (1:5,000), anti-N-DnaJ (1:200), anti-C-DnaJ (1:500), anti-DNAJA1 (1:500), and anti-DNAJA2 (1:500), for 1 h at room temperature. The secondary antibodies were used at 1:200 dilutions.

### ELISA assays

ELISA tests were performed as described by Krzewski et al. (2003), with minor modifications. Costar 3590 96-well plates were coated with serial dilutions of DnaJ, N-DnaJ, C-DnaJ, DNAJA1, or DNAJA2 proteins (40–0.02 μg/mL) and incubated overnight at 37 °C. Then, 50 μL of a diluted anti-DnaJ mAb was added to the wells. The mAbs were used at the following dilutions: AC11—1:6,000; BB3, CC8, and EE11—1:2,000; DC7—1:500; and CC8—1:200. After 60 min of incubation, the secondary antibodies coupled with horseradish peroxidase (HRP) (Sigma) were added: the goat anti-mouse IgG antibodies to AC11 and the goat anti-mouse IgM antibodies to the other mAbs, at 1:2,000 dilutions. The bound conjugates were detected colorimetrically after 60 min of incubation, by using a horseradish peroxidase substrate, tetramethylbenzidine (Sigma).

When the reactivity of the Hsp40 proteins with the rabbit polyclonal anti-Hsp40 sera was tested, the sera were used at the following dilutions: anti-DnaJ (1:40,000), anti-N-DnaJ (1:1,000), anti-C-DnaJ (1:4,000), anti-DNAJA1 (1:4,000), and anti-DNAJA2 (1:4,000). HRP-conjugated goat anti-rabbit IgG immunoglobulins (Dako) (1:2,000) were used as secondary antibodies.

The levels of the anti-DnaJ, anti-DNAJ1, and anti-DNAJ2 antibodies (IgG) in the sera of the RA patients (*n* = 43) and the control individuals (*n* = 35) were assayed by the ELISA test performed as described before (Tukaj et al. 2010b). The antigens used were the Hsp40 proteins produced in the bacterial cells and, additionally, the DNAJA1 and DNAJA2 produced and farnesylated in the baculovirus system (designated as DNAJA1^f^ and DNAJA2^f^).

### The RA patients

The RA patients’ sera were obtained from 43 (29 females and 14 males) patients (the mean age was 59, range 21–82), with the early RA (the mean disease duration was 9 months). For controls, we used sera of 35 sex- and age-matched healthy volunteers. The study was approved by the Local Committee for Biomedical Research Ethics at the Medical University of Gdansk. All subjects were informed of the details of the experiment prior to the taking of a sample of 10 mL peripheral venous blood.

### Statistical analysis

Statistical analyses were performed using the Mann–Whitney *U* test and STATISTICA 7.1 software. For correlation analyses, Pearson test was used. *p* values less than 0.05 were considered significant.

## Electronic supplementary material

Below is the link to the electronic supplementary material.

ESM 1 (DOC 47.5 kb)
