# Supplementary material for: Human Hsp40 proteins, DNAJA1 and DNAJA2, as potential targets of the immune response triggered by bacterial DnaJ in rheumatoid arthritis
Source: Cell Stress Chaperones. 2013 Feb 14;18(5):653–9. doi: 10.1007/s12192-013-0407-1 (PMC3745263; doi:10.1007/s12192-013-0407-1)
Supplement: Supplementary file 1 — (DOC 47.5 kb) [file 12192_2013_407_MOESM1_ESM.doc]

| Antibody titer | DnaJ | DNAJA1 | DNAJA2 | DNAJA1^f^ | DNAJA2^f^ |
| --- | --- | --- | --- | --- | --- |
| RA vs.  Control (2.5) | 0.336 ± 0.16 vs. 0.086 ± 0.02 ^***^ | 0.226 ± 0.13 vs.  0.064 ± 0.02 ^***^ | 0.183 ± 0.079 vs.  0.075 ± 0.033 ^***^ | 0.257 ± 0.06 vs.  0.040 ± 0.01 ^***^ | 0.123 ± 0.06 vs.  0.041± 0.02 ^***^ |
| RA vs.  Control (0.15) | 0.102 ± 0.054 vs.  0.041 ± 0.019 ^***^ | 0.079 ± 0.05 vs.  0.038 ± 0.019^***^ | 0.063 ± 0.024 vs.  0.036 ± 0.015 ^***^ | 0.050 ± 0.018 vs.  0.013 ± 0.004 ^***^ | 0.022 ± 0.007 vs.  0.014 ± 0.006 ^***^ |

Supplementary data

Table S1. The levels of the antibodies against the Hsp40 proteins in the sera of RA patients and healthy controls

The levels of the antibodies were assayed by ELISA test in the sera of 43 RA patients and 35 healthy controls as described in “Materials and methods”, using antigens at the concentrations (μg/mL) indicated in parentheses. The DNAJA1^f^ and DNAJA2^f^  are the farnesylated proteins, produced in the baculovirus system. The other proteins were produced in the bacterial system. The values are expressed as mean ±SD of Absorbance _450/620_. The statistically significant values are indicated by the asterisks. To assay significance of the difference between the RA and control antibody levels *U* Mann–Whitney test was used. ^***^ P<0.001.
